# Recent advances in photocatalyzed reactions using well-defined copper(I) complexes

**DOI:** 10.3762/bjoc.16.42

**Published:** 2020-03-23

**Authors:** Mingbing Zhong, Xavier Pannecoucke, Philippe Jubault, Thomas Poisson

**Affiliations:** 1 Normandie Université, INSA Rouen, UNIROUEN, CNRS, COBRA (UMR 6014), 76000 Rouen, France; 2 Institut Universitaire de France, 1 rue Descartes, 75231 Paris, France

**Keywords:** ATRA reactions, copper catalysis, energy transfer, oxidation, PCET reactions, photocatalysis, reduction

## Abstract

This review summarizes the recent advances in photocatalysis using copper complexes. Their applications in various reactions, such as ATRA, reduction, oxidation, proton-coupled electron transfer, and energy transfer reactions are discussed.

## Introduction

For a decade, the realm of catalysis has known a significant renewal with the rise of photocatalysis [1]. The fact that a reaction can be carried out, and, more specifically, catalyzed in the presence of visible light and a photosensitive catalyst (organic or organometallic) provided a drastic change of paradigm to the community. Indeed, photocatalysis allows carrying out photochemical reactions in the visible region (redox transformation and energy transfer process). This tremendous progress provided a huge gain of selectivity in photochemical transformations. Indeed, as most organic molecules absorb light in the UV region (UV-A, UV-B, or UV-C), selectivity was a key point to address in photochemistry and photocatalysis using visible light tackled this issue. Moreover, the use of inexpensive and energy-efficient visible light-emitting diodes (LEDs) was an impressive development. Furthermore, photocatalysis allowed to realize elusive transformations as this approach provided better control over the formation of radicals or the reduction or oxidation of reagents, for instance. For all these reasons, photocatalysis is nowadays at the forefront of cutting-edge research in organic chemistry.

Despite the impressive advances reported since the renewal of the field in 2008 [2–4], several issues still have to be addressed. Indeed, most of the developed reactions relied on the use of organometallic complexes of expensive noble metals, such as iridium and ruthenium [5]. Even though their efficiency is outstanding, their use for industrial purposes might be hampered. Indeed, the limited availability of these metals and their prices often prohibit the development of economically efficient chemical processes [6]. In addition, the depletion of natural resources raises questions about the future availability of these metals at a reasonable cost. With regards to organic dyes, impressive developments have been achieved, but these catalysts might suffer from a low photochemical stability, and thus hampering their use and recyclability [7]. Therefore, alternative solutions have to be developed. Among them, the use of first-row transition metals [8–9], particularly copper, is an interesting approach [10–13]. The high abundance, low price, low toxicity, and intrinsic properties (redox potential, four oxidation states, etc.) of copper are excellent and promising features to be used in the development of new photocatalysts. However, the study, development, and use of these complexes have been underexplored since the pioneering work of McMillin [14] and Sauvage [15], compared to the tremendous expansion of photocatalyzed reactions using Ru and Ir complexes. Indeed, copper-based photocatalysts are often considered less efficient due to a lower luminescence lifetime compared to Ir and Ru complexes [6].

## Review

In this review, we summarized the recent significant advances that were made in the use of copper-based photocatalysts for synthetically useful transformations. The use of either homoleptic or heteroleptic complexes in atom transfer radical addition (ATRA) reactions, reductions, oxidations, proton-coupled electron transfer (PCET) reactions, and reactions based on energy transfer will be discussed.

### 1 Homoleptic Cu(I) complexes

Homoleptic complexes based on bisnitrogen ligands, and particularly phenanthroline derivatives, were used in the pioneering reports from McMillin [14] and Sauvage [15]. For decades, their use in organic synthesis, and particularly catalysis, has been ignored. However, since 2012, a renewed interest toward their use in catalysis has been witnessed.

#### 1.1 ATRA reactions

Atom transfer radical addition reactions are linchpin transformations in organic synthesis as they allow an easy difunctionalization of alkenes. Usually, these reactions require the use of a radical initiator or thermal activation to initiate the radical chain. Recently, photocatalysis appeared as an interesting alternative to catalyze such transformations, and copper-based catalysts provided interesting reactivities.

**Scheme 1:**
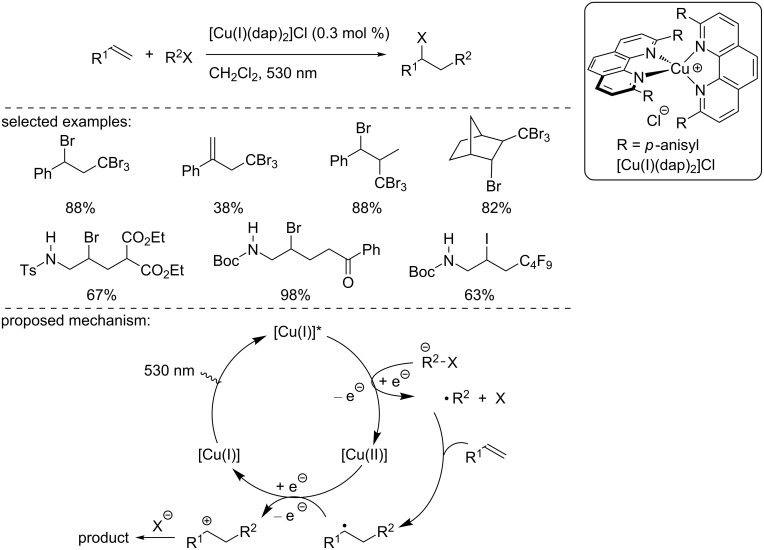
[Cu(I)(dap)_2_]Cl-catalyzed ATRA reaction under green light irradiation.

In 2012, the Reiser group reported a visible light-driven coupling reaction of olefin derivatives with bromo- and iodoalkanes using the Sauvage catalyst as a Cu(I) photocatalyst (Scheme 1) [16]. The [Cu(I)(dap)_2_]Cl complex had a strong absorption under irradiation at 530 nm using a green LED. Different organic halides and alkenes were reacted, leading to the product by an ATRA reaction pathway with moderate to good yield at room temperature (Scheme 1). The authors suggested a possible mechanism based on the measured and reported redox potential (Scheme 1).

Upon irradiation at 530 nm using green light, the Cu(I) catalyst transitions to an excited state. Then, the excited copper complex transfers an electron to the alkyl halide, which can generate an alkyl radical that subsequently adds to the alkene. The resulting C-centered radical is oxidized by the Cu(II) complex, regenerating the Cu(I) catalyst, and the formed carbocation is trapped by the halide. Worth to mention is that very recently, Reiser and Engl demonstrated the possible use of [Cu(dmp)_2_Cl]Cl as an efficient catalysts in a similar transformation [17].

**Scheme 2:**
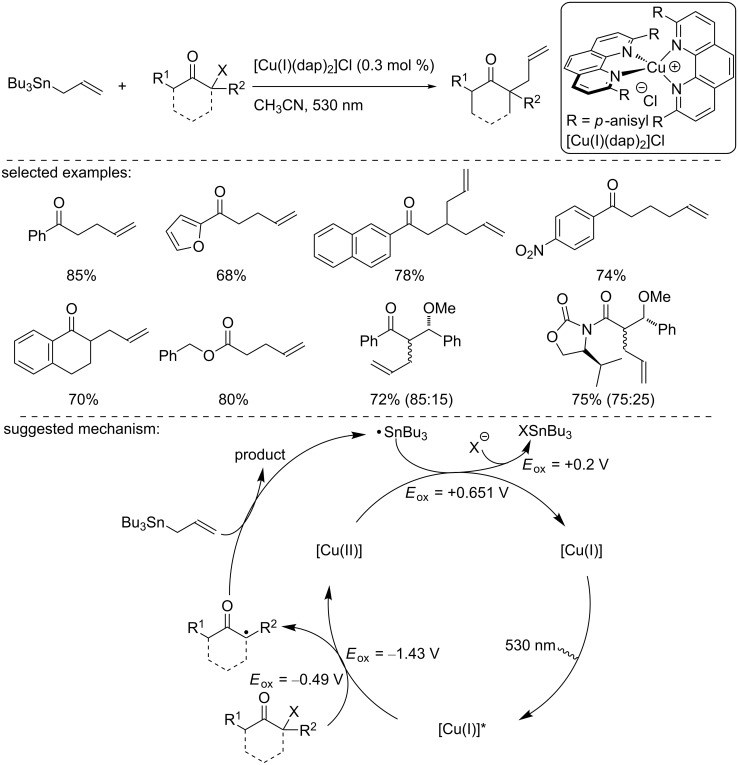
Photocatalytic allylation of α-haloketones.

In another study, Reiser and co-workers described a related allylation process of organic halides with allyltributyltin in the presence of [Cu(I)(dap)_2_]Cl as the catalyst [16]. This reaction was applied to a broad range of substrates, including a *para*-nitrophenyl ketones and furanyl ketones, for instance, with good yield. To explain the reaction outcome, the authors suggested that the [Cu(I)(dap)_2_]Cl catalyst acted as an electron shuttle between the halide derivative and the allylmetal reagent, precluding a direct electron transfer between the allylstannane and the haloketone. Hence, after being excited by light, the excited [Cu(I)]* complex gave an electron to the α-haloketone. Then, the ketone radical combined with allyltributyltin to generate a Bu_3_Sn radical. A final electron transfer from the Bu_3_Sn radical to [Cu(II)] regenerated the Cu(I) catalyst (Scheme 2).

**Scheme 3:**
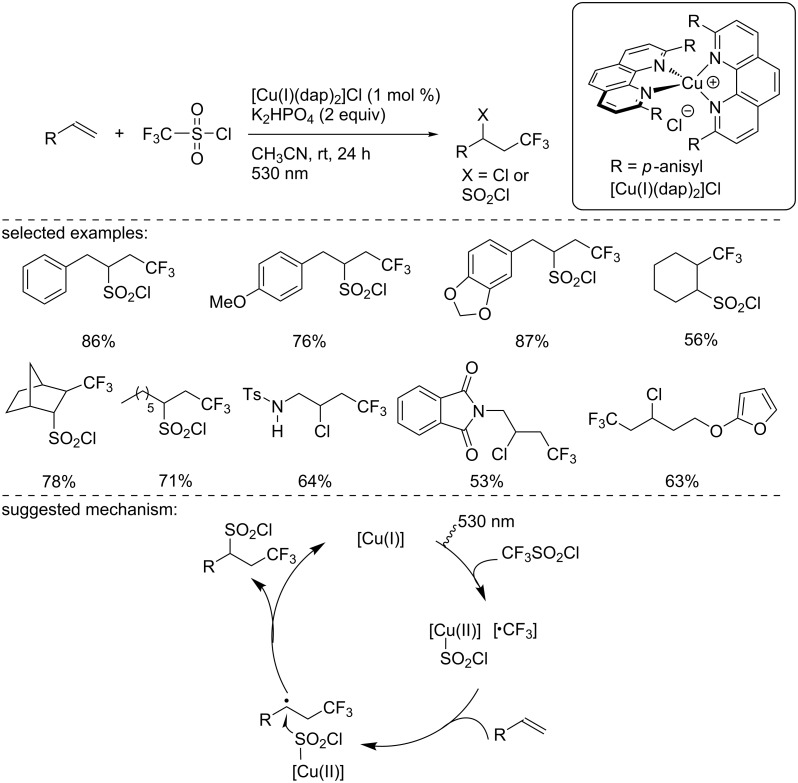
[Cu(I)(dap)_2_]Cl-photocatalyzed chlorosulfonylation and chlorotrifluoromethylation of alkenes.

Based on this work, in 2015, Reiser and co-workers reported another ATRA reaction to carry out the trifluoromethylchlorosulfonylation of alkenes (Scheme 3) [18]. They used the same catalyst as above, [Cu(I)(dap)_2_]Cl, to introduce the CF_3_ group to different alkenes under green LED irradiation (530 nm), with CF_3_SO_2_Cl as the CF_3_ source. Depending on the substrates, both chlorosulfonylated and chlorotrifluoromethylated products could be obtained. For allylbenzene as well as terminal and cyclic alkenes, the trifluoromethylchlorosulfonylated adducts were isolated in good yield and selectivity. When an electron-donating group (e.g., NH, NHCO, and NMe_2_) was in close proximity to the alkene, the pathway of the addition changed and the chlorotrifluoromethylated adduct was formed. To explain the reaction mechanism, the authors suggested the following pathway: Upon irradiation at 530 nm using green light, the Cu(I) catalyst transitions to the excited state. Then, the excited copper complex undergoes an electron transfer to CF_3_SO_2_Cl, allowing the formation of the CF_3_ radical, and the resulting Cu(II) species binds to the SO_2_Cl anion, known to be unstable when free. Then, the CF_3_ radical adds to the alkene, followed by the addition of SO_2_Cl to produce the desired product. However, when the reaction rate is slower, the SO_2_Cl anion decomposes to neutral SO_2_ and a chloride anion due to the weak nature of the Cu–SO_2_Cl bond. The SO_2_ extrusion explains the formation of the chlorotrifluoromethylated product (Scheme 3).

In 2019, Bissember and co-workers reported the synthesis of new homoleptic copper complexes by modifying the structure of the phenanthroline ligand at the 1- and 10-positions with various aromatic substituents [19]. These complexes were fully characterized and evaluated in a similar reaction. They observed that the variation of the electronic and steric parameters of the ligand could modulate the selectivity toward the chlorotrifluoromethylated and chlorosulfonylated products.

**Scheme 4:**
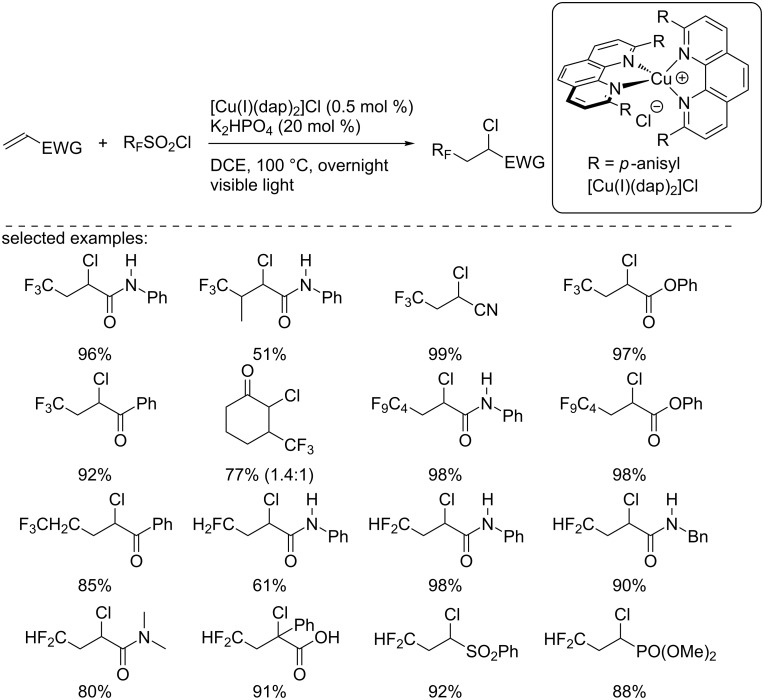
Photocatalytic perfluoroalkylchlorination of electron-deficient alkenes using the Sauvage catalyst.

Earlier, in 2015, the group of Dolbier had reported a similar reaction with fluoroalkylsulfonyl chlorides [20]. This ATRA reaction was carried out with various fluoroalkylsulfonyl chlorides, such as CFH_2_SO_2_Cl, CF_2_HSO_2_Cl, CF_3_SO_2_Cl, CF_3_CH_2_SO_2_Cl, and C_4_F_9_SO_2_Cl, electron-deficient alkenes, including α,β-unsaturated ketones, amides, esters, carboxylic acids, sulfones, and phosphonates (Scheme 4). In contrast to Reiser’s protocol, solely chlorofluoroalkylated products were isolated in good yields, and no traces of the chlorosulfonylated alkenes were detected.

**Scheme 5:**
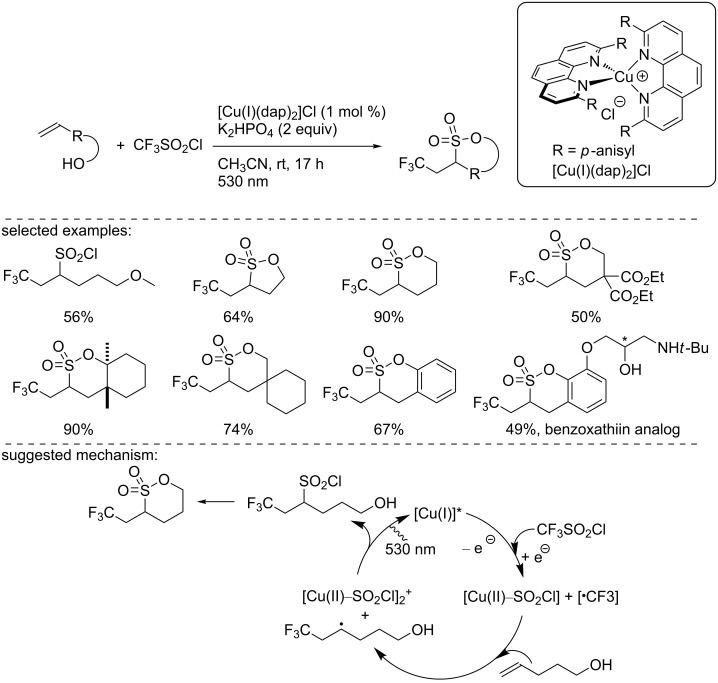
Photocatalytic synthesis of fluorinated sultones.

In 2016, Reiser’s group used a similar protocol to synthesize trifluoromethylated sultones from alkenol and sulfonyl chlorides with the catalyst [Cu(I)(dap)_2_]Cl under green light irradiation (Scheme 5) [21]. Fluorinated sultones are important scaffolds for pharmaceutical syntheses. To illustrate the synthetic utility of the method, a novel benzoxathiin analog was synthesized using this reaction as the key step. The authors suggested a similar radical pathway as the one described previously (Scheme 3). This mechanism involved the formation of a CF_3_ radical through a single-electron transfer between the excited complex [Cu(I)(dap)_2_]^+^* and triflyl chloride. This radical added to the alkene, and the SO_2_Cl anion coordinated to the [Cu(II)] species. Upon electron back transfer to regenerate the [Cu(I)] catalyst, SO_2_Cl was also incorporated into the product. Finally, an intramolecular cyclization process gave the desired product (Scheme 5).

**Scheme 6:**
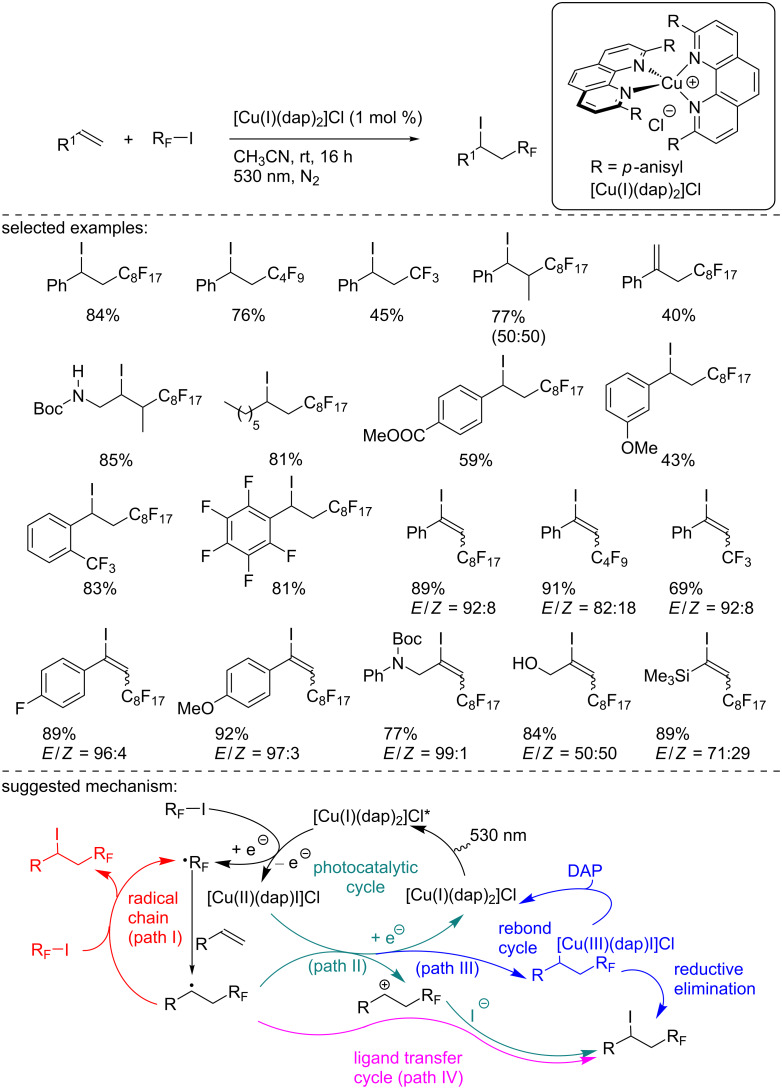
Photocatalyzed haloperfluoroalkylation of alkenes and alkynes.

Two years later, Reiser’s group reported another ATRA reaction of perfluoroalkyl iodides with alkenes and alkynes. [Cu(I)(dap)_2_]Cl was found to be the best catalyst for this transformation proceeding upon irradiation at 530 nm (green LED, Scheme 6) [22]. Indeed, the comparison of this Cu catalyst with [Ru(bpy)_3_]Cl_2_ and Ir(ppy)_3_ clearly demonstrated the higher efficiency, good yield, selectivity, and excellent functional group tolerance of the method. Further, the authors carried out an interesting mechanistic study: From their observations, they ruled out a possible radical chain process (path I) because the reaction with styrene cannot be initiated by heating or through a radical initiator. In addition, the authors precluded a possible photocatalytic process (path II). And indeed, even though from a comparison of the redox potential of [Cu(I)(dap)_2_]Cl and [Ru(bpy)_3_]Cl_2_, the latter should have been capable to promote the photocatalytic reaction, the ruthenium complex was unsuccessful. Hence, to explain the reaction outcome, the authors suggested two possible pathways: First, a common reductive SET between the [Cu(I)] species and the perfluorinated iodoalkane, affording the perfluorinated C-centered radical and the [Cu(II)] complex [Cu(II)(dap)I]Cl. The perfluorinated radical then adds to the alkene to form a new C-centered radical. The first possible pathway relied on a rebound cycle where this radical recombined with the [Cu(II)] complex to generate a [Cu(III)] species. Then, a reductive elimination closes the catalytic cycle, delivering the product and regenerating the catalyst, along with an exchange of the ligand iodide for DAP (path III). Another possibility was the trapping of the radical with the iodine ligand of the [Cu(II)] catalyst (path IV).

**Scheme 7:**
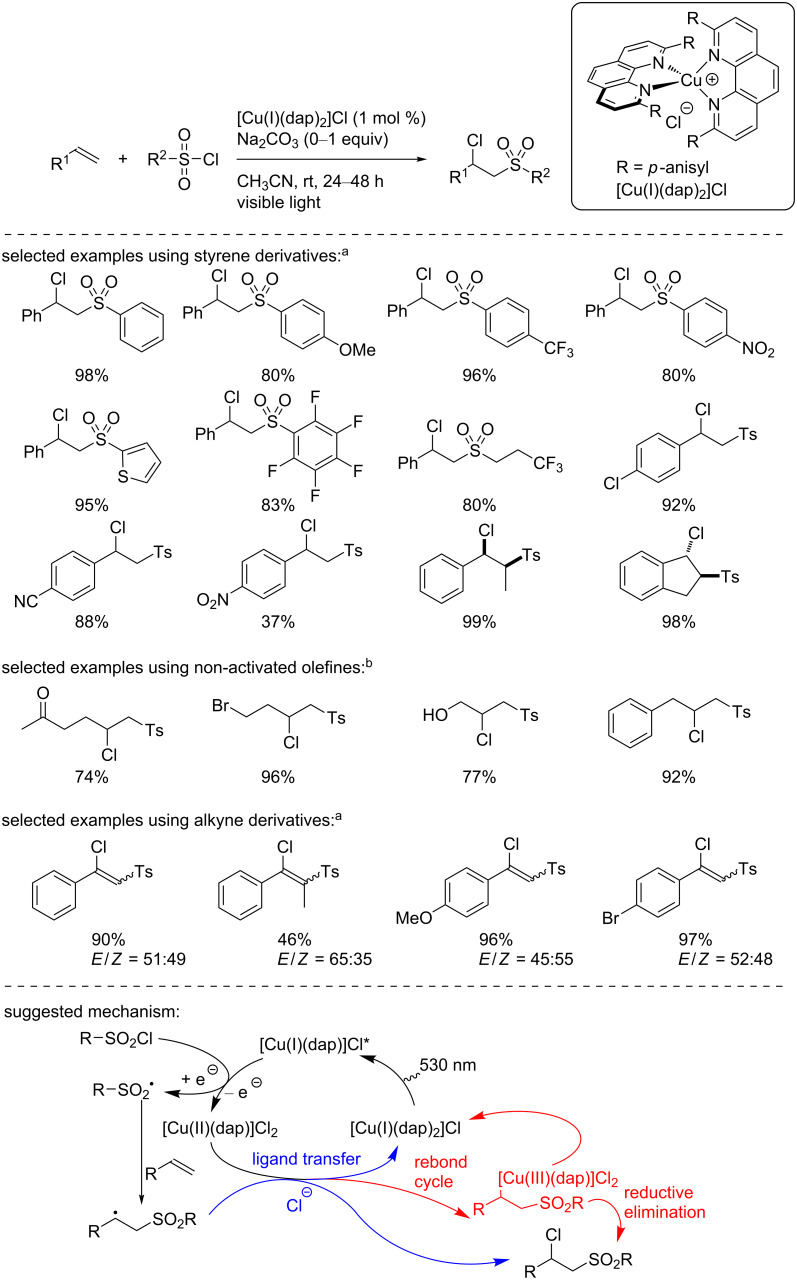
Chlorosulfonylation of alkenes catalyzed by [Cu(I)(dap)_2_]Cl. ^a^No Na_2_CO_3_ was added. ^b^1 equiv of Na_2_CO_3_ was used.

In 2019, Reiser’s group described a chlorosulfonylation reaction of alkenes and alkynes with [Cu(I)(dap)_2_]Cl or [Cu(II)(dap)_2_]Cl_2_ under visible light irradiation (Scheme 7) [23]. The scope of this transformation was excellent, demonstrating good functional group tolerance and the products were obtained in good to excellent yields. Note that the reaction yields were similar or even sometimes higher when the [Cu(II)(dap)_2_]Cl_2_ complex was used as a catalyst. To explain the reaction outcome, the authors suggested the involvement of a [Cu(I)] complex as the catalytically active species, even when [Cu(II)(dap)_2_]Cl_2_ was used. Indeed, they suggested the formation of the [Cu(I)] species from a homolytic cleavage of the Cu(II)–Cl bond under light irradiation [24]. First, the excited [Cu(I)]* complex is involved in an SET with the sulfonyl chloride, forming the sulfonyl radical and a [Cu(II)] species binding a chloride anion. The sulfonyl radical stemming from this reduction can add to the alkene, forming a C-centered radical. Then, two plausible pathways were suggested. First, a chlorine ligand transfer to the C-centered radical can proceed, delivering the product and producing the [Cu(I)] species in the ground state. Another possible pathway focused on a rebound between the [Cu(II)] species and the C-centered radical to form a [Cu(III)] species that can deliver the product through a reductive elimination, along with the [Cu(I)] species in the ground state.

#### 1.2 Reduction reactions

**Scheme 8:**
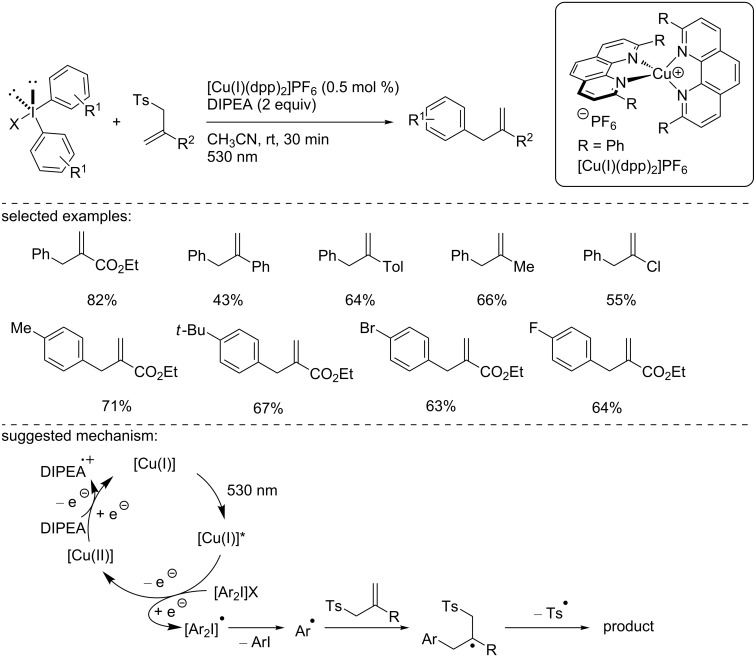
Copper-photocatalyzed reductive allylation of diaryliodonium salts.

In 2013, Fensterbank, Goddard, and Ollivier reported the use of the homoleptic complex [Cu(I)(dpp)_2_]PF_6_ for the reduction of symmetrical diaryliodonium salts (Scheme 8) [25]. This copper-catalyzed photocatalytic reduction generated an aryl radical that was trapped with various allylating reagents. First, the phenyl radical generated from the corresponding diphenyliodonium salt was added to various allyl sulfones substituted in the 2-position. The products were isolated in moderate to good yields. Using the more reactive allyl sulfone, a panel of symmetrical diaryliodonium salts was reacted, giving the products in good yields. The authors conducted some mechanistic studies and suggested a plausible catalytic cycle involving [Cu(I)]/[Cu(I)]*/[Cu(II)] species. The [Cu(I)(dpp)_2_]PF_6_ complex can be excited under visible light irradiation (530 nm), and the resulting excited [Cu(I)]* complex undergoes an SET to reduce the diaryliodonium species, providing the oxidized [Cu(II)] complex. The reduced diaryliodonium species collapses into an aryl radical and the corresponding aryl iodide. The aryl radical can then add to the allylating reagent, which, after tosyl radical elimination, provides the desired product. Finally, the active catalyst is regenerated thanks to the use of DIPEA as a sacrificial reductant. Note that this reaction was inefficient with aryl iodides, and no significant selectivity was observed when nonsymmetrical iodonium salts were reacted under the optimized reaction conditions.

**Scheme 9:**
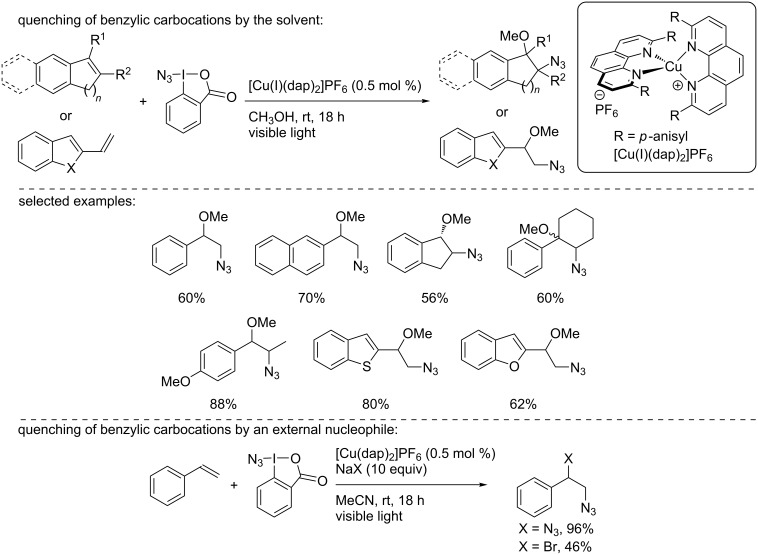
Copper-photocatalyzed azidomethoxylation of olefins.

Later in 2015, Greaney and co-workers described the copper-photocatalyzed azidation of styrene olefins and the concomitant introduction of a nucleophile (Scheme 9) [26]. Using the Zhdankin reagent as the azidyl radical precursor and the Sauvage catalyst [Cu(I)(dap)_2_]Cl in methanol, various styrenes were readily converted into the azidomethoxylated products in good to excellent yields. The reaction was also extended to α- and β-substituted styrenes, cyclic alkenes, and heteroaromatic derivatives. Noteworthy, when the reaction was carried out in acetonitrile, azido bromination and diazidation reactions were possible using 10 equivalents of NaBr and NaN_3_, respectively. Notably, when the reaction was carried out in the dark, the diazidation reaction of the olefins occurred, while the use of Ir or Ru complexes in this transformation led to the degradation of the reagents. As this process did not involve a photocatalytic pathway, it will not be discussed here. To explain the reaction outcome, based on literature data, the authors hypothesized a catalytic cycle involving [Cu(I)]/[Cu(I)]*/[Cu(II)] species and the reduction of the Zhdankin reagent by the copper catalyst to form an azidyl radical, which then reacted with the olefin. The resulting benzyl radical could then be oxidized, probably by the catalyst in the +II oxidation state, to generate a benzylic carbocation and the active [Cu(I)] catalyst. Finally, the solvent or the nucleophile introduced to the reaction medium reacted with the latter.

**Scheme 10:**
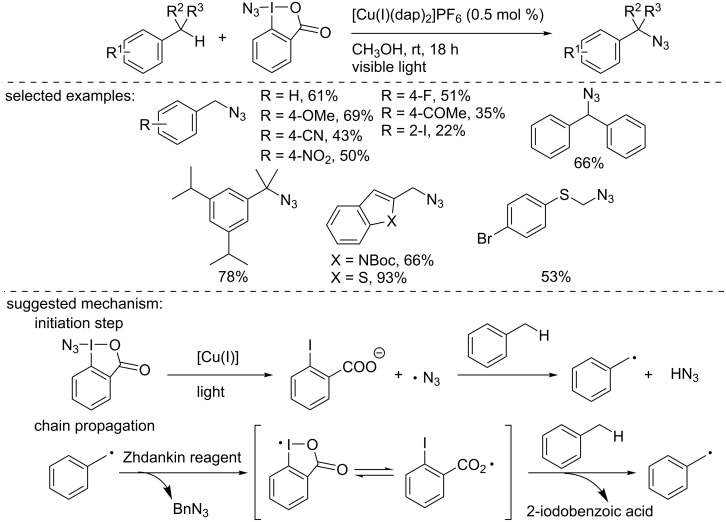
Benzylic azidation initiated by [Cu(I)(dap)_2_]Cl.

Later, Greaney and co-workers reported the photocatalytic azidation of benzylic C–H bonds (Scheme 10) [27]. Using the Sauvage catalyst [Cu(I)(dap)_2_]PF_6_ and the Zhdankin reagent, a large panel of substrates was functionalized under visible light irradiation. The functional group tolerance of the process was excellent and the products were obtained in moderate to excellent isolated yields. The authors suggested a chain radical process to explain the formation of the product. The copper photocatalyst initiated the formation of the azidyl radical, which abstracted the benzylic hydrogen atom from the substrate. Then, the benzylic radical reacted with the Zhdankin reagent, producing the azidated product and propagating the radical chain through the reaction of the iodane radical with the benzylic substrate.

**Scheme 11:**
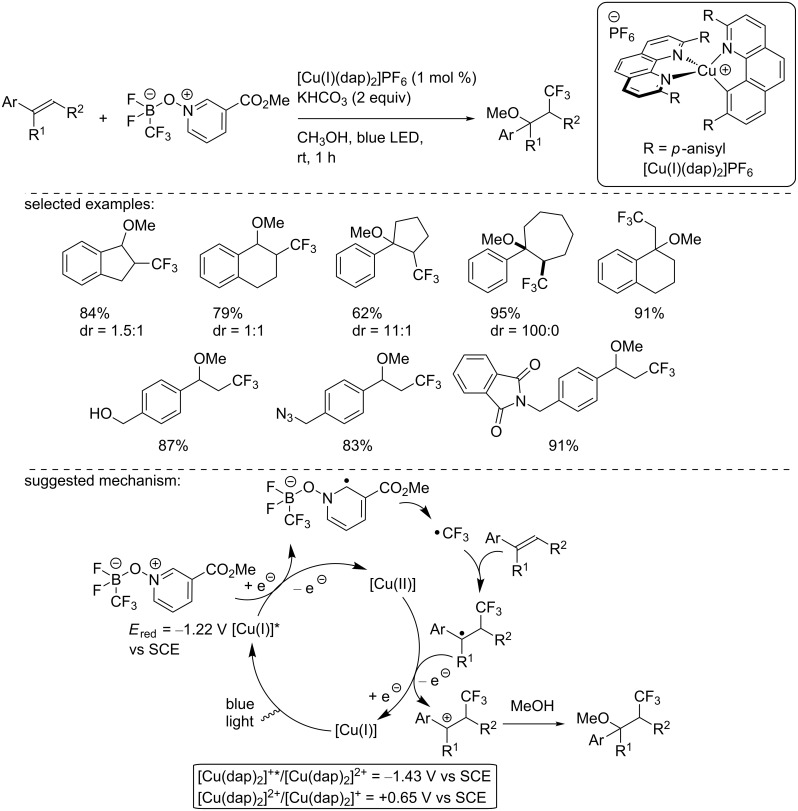
Trifluoromethyl methoxylation of styryl derivatives using [Cu(I)(dap)_2_]PF_6_. All redox potentials are reported vs SCE.

In 2018, Dilman and co-workers reported the catalytic formation of the trifluoromethyl radical through the photocatalytic reduction of a borate complex (Scheme 11) [28]. To carry out this reduction, the authors used the PF_6_ salt of the Sauvage catalyst under blue LED irradiation in MeOH to perform the trifluoromethyl methoxylation of styrene derivatives. The methodology was applied to a broad range of styrene derivatives, showing a good functional group tolerance. Noteworthy, α-, β-, and α,β-substituted styrenes were readily functionalized in good to excellent yields. To explain the reaction outcome, the authors suggested a reduction of the trifluoromethyl borate complex according to an SET with the excited copper(I) complex. The resulting substituted pyridyl radical eliminated a trifluoromethyl radical, which then reacted with the alkene. Then, the formed benzylic radical was oxidized to the corresponding carbocation, regenerating the photocatalyst in the ground state. The benzylic carbocation was finally trapped with MeOH, which was used as the solvent to form the trifluoromethyl methoxylated product.

**Scheme 12:**
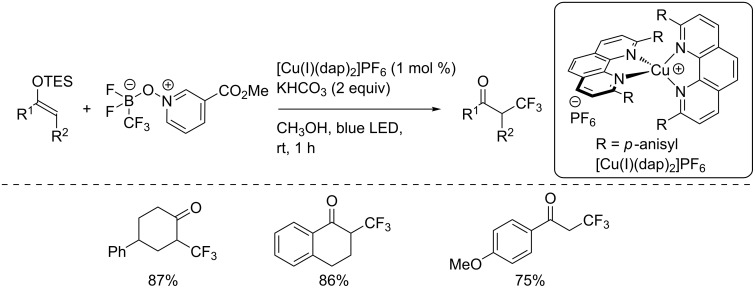
Trifluoromethylation of silyl enol ethers.

In the same publication, Dilman and co-workers reported the addition of a trifluoromethyl radical to silyl enol ethers derived from ketones using the same reaction conditions (Scheme 12) [28].

#### 1.3 Oxidation reactions

**Scheme 13:**
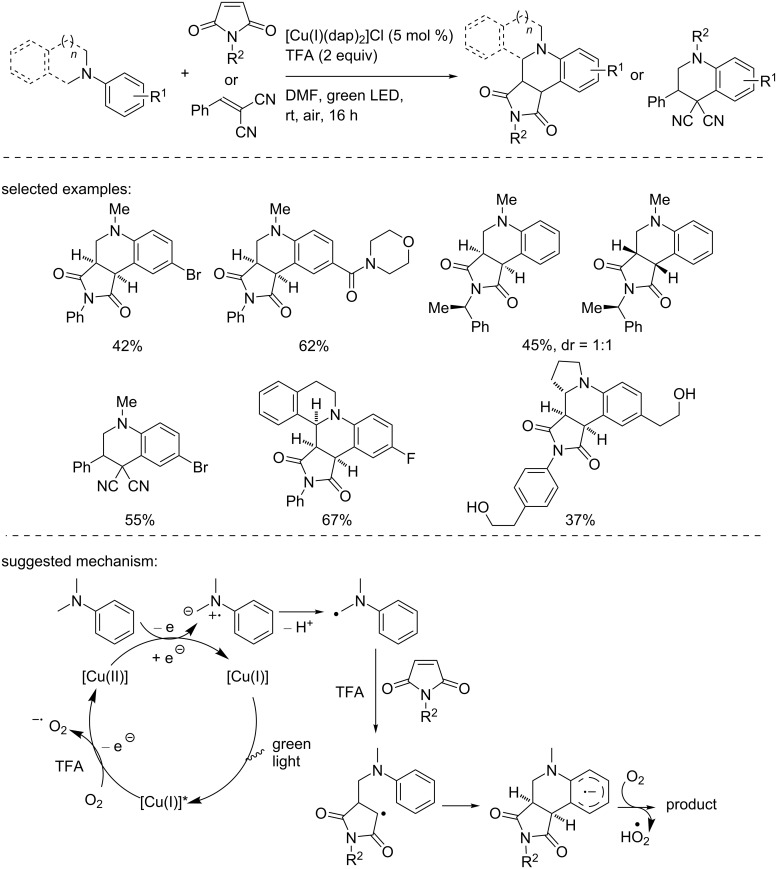
Synthesis of annulated heterocycles upon oxidation with the Sauvage catalyst.

In 2015, Bissember and co-workers used the Sauvage catalyst to generate an α-amino radical, which was used to perform the synthesis of annulated tetrahydroquinolines and octahydroisoquinolo[2,1-*a*]pyrrolo[3,4-*c*]quinolines (Scheme 13) [29]. Importantly, the formation of the key α-amino radical resulted from an oxidation reaction catalyzed by the copper catalyst in the oxidation state +II. Using the [Cu(I)(dap)_2_]Cl complex as the catalyst and 2 equivalents of TFA under air, *N*,*N*-dialkylated aniline derivatives were reacted with various *N*-substituted maleimides and benzylidenemalonitrile to provide polysubstituted tetrahydroquinolines in moderate to good yields. When *N*-aryltetrahydroisoquinoline was used instead of *N*,*N*-dialkylated anilines, octahydroisoquinolo[2,1-*a*]pyrrolo[3,4-*c*]quinoline derivatives were obtained for the first time.

This transformation starts with the oxidation of the excited photocatalyst with O_2_. The aniline is then oxidized into an N-centered radical cation, which further gives the α-amino radical. The latter reacts with the maleimide to provide a transient radical, which undergoes an intramolecular cyclization to give the aryl radical anion. A final oxidation/deprotonation sequence delivers the product.

**Scheme 14:**
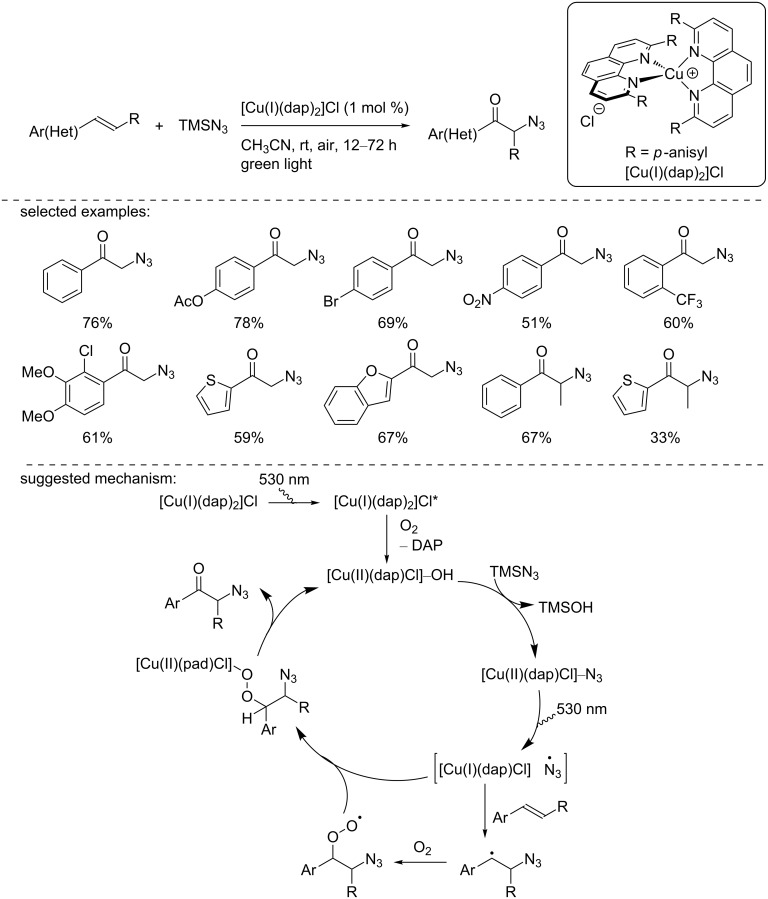
Oxoazidation of styrene derivatives using [Cu(dap)_2_]Cl as a precatalyst.

In 2018, Reiser and co-workers reported the use of the [Cu(dap)_2_]Cl catalyst in the oxoazidation of styrene derivatives (Scheme 14) [30]. Under green light irradiation, a large panel of styrene derivatives was converted into the corresponding α-azidoketones in good to excellent yields. Interestingly, the reaction was extended to the heteroaromatic derivatives, such as thiophene or benzofuran and to β-substituted styrenes. The authors conducted mechanistic studies and demonstrated the role of [Cu(dap)_2_]Cl as a precatalyst since the catalytically active species was a [Cu(II)] complex. To explain the formation of the product, the authors suggested that after excitation, the [Cu(I)] catalyst is oxidized to a [Cu(II)] species along with the loss of a DAP ligand. Then, the [Cu(II)] complex reacts with TMSN_3_ to form a [Cu(II)]–N_3_ species that gives an azide radical through a homolytic dissociation induced by light irradiation. The formed azide radical reacts with the styrene to form a benzylic radical that is then oxidized by O_2_. The resulting peroxyl radical reacts with the Cu complex, forming a new [Cu(II)] species that collapses to deliver the product and regenerates the active [Cu(II)] catalyst.

### 2 Heteroleptic Cu(I) complexes

Heteroleptic copper complexes appeared as an interesting and promising alternative to the homoleptic examples. Indeed, they usually possess an increased lifetime of the triplet excited state due to a particular geometry that prevents the reorganization from a tetrahedral geometry in the ground state to a square planar geometry [31]. Moreover, the synthesis of these compounds is usually facile, and they can be readily obtained in a crystalline form. Herein, we will report their use in ATRA reactions, reductions, oxidations, as well as in PCET and energy transfer reactions.

#### 2.1 ATRA reactions

**Scheme 15:**
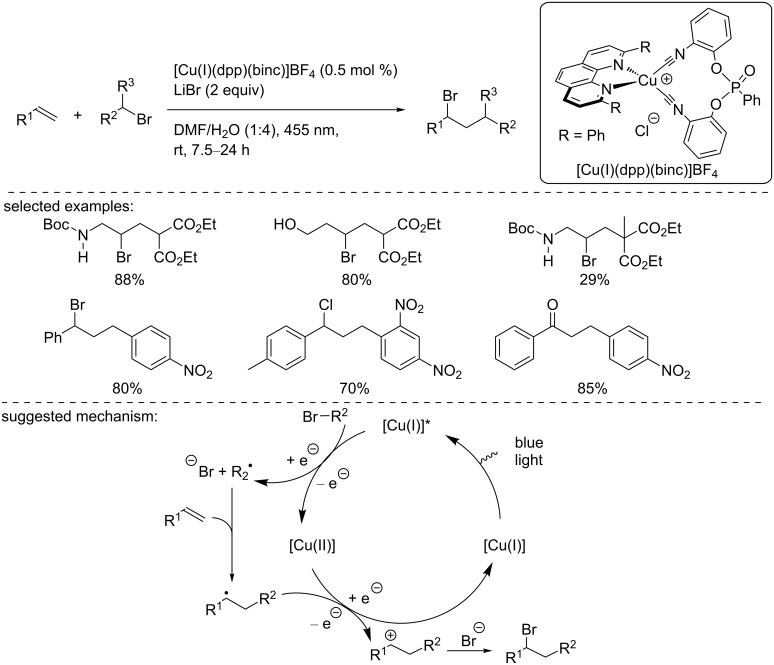
[Cu(I)(dpp)(binc)]PF_6_-catalyzed ATRA reaction.

In 2015, Reiser and co-workers reported the synthesis of a novel copper-based heteroleptic complex bearing a phenanthroline derivative and a bisisonitrile ligand, [Cu(I)(dpp)(binc)]BF_4_, which was fully characterized (Scheme 15) [32]. This complex proved to have a similar or higher excited state lifetime (17 μs) compared to the previously reported complexes and a redox potential of −1.88 V vs SCE in the excited state ([Cu(I)]*/[Cu(II)]). Upon irradiation at 455 nm, the authors used this complex in ATRA reactions, with various ATRA reagents, including α-bromomalonates and benzyl bromides, in combination with a broad range of alkenes (allylamine, homoallyl alcohol, styrene derivatives, and silyl enol ethers). The corresponding products were isolated in moderate to excellent yields. The authors suggested a classical mechanism for this ATRA. First, the excited copper complex reduced the organohalide to form a C-centered radical. The latter reacted with the olefin to form a new carbon-centered radical. Then, the catalyst in the oxidation state +II oxidized the newly formed radical to the corresponding carbocation along with the regeneration of the catalyst in the ground state. Finally, the carbocation was trapped by the anion generated in the first reduction step.

**Scheme 16:**
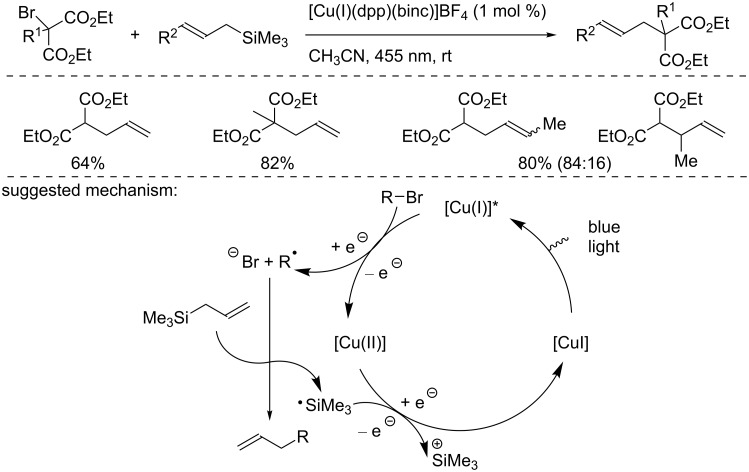
Allylation reaction of α-bromomalonate catalyzed by [Cu(I)(dpp)(binc)]PF_6_ following an ATRA mechanism.

In the same report, Reiser and co-workers reported an allylation reaction using the [Cu(I)(dpp)(binc)]BF_4_ complex (Scheme 16) [32]. The reaction of α-bromomalonates with allyl- and crotylsilanes afforded the allylated products in good yields. To explain the reaction outcome, the authors suggested a reduction of the α-bromomalonate by the excited Cu(I) complex. Then, the generated radical adds to the allylsilane, releasing a silyl radical. Then, the latter oxidizes the Cu(II) complex and regenerates the catalyst in the ground state.

**Scheme 17:**
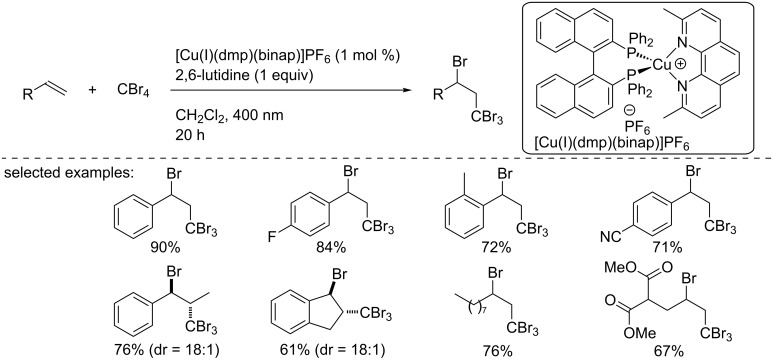
Bromo/tribromomethylation reaction using [Cu(I)(dmp)(BINAP)]PF_6_.

In 2018, Yamaguchi and Itoh reported the ATRA of CBr_4_ to various alkenes, including styrene derivatives and aliphatic-substituted olefins (Scheme 17) [33]. The reaction was carried out using the complex [Cu(I)(dmp)(binap)]PF_6_, and the desired tribromomethylated products were isolated in good to excellent yields. The authors suggested a similar mechanism to the one described by Reiser and co-workers in Scheme 14.

**Scheme 18:**
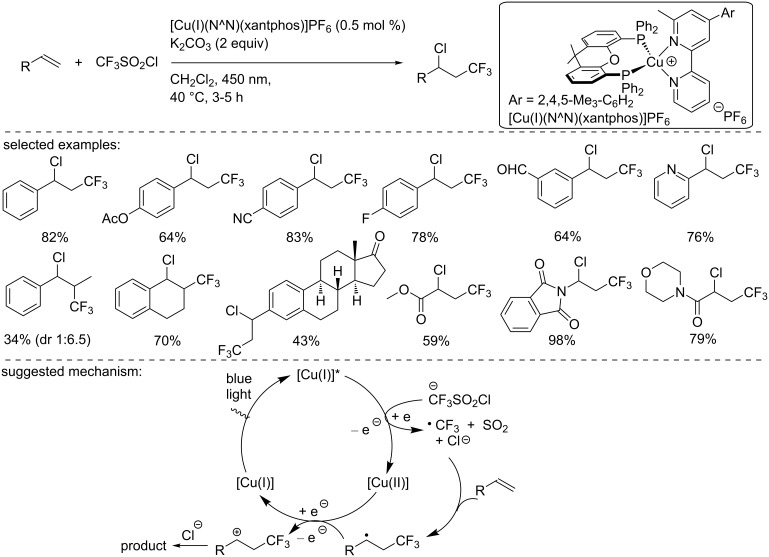
Chlorotrifluoromethylation of alkenes catalyzed by [Cu(I)(N^N)(xantphos)]PF_6_.

In 2018, Hu and co-worker described the synthesis of a new heteroleptic copper complex bearing a substituted bipyridine ligand (Scheme 18) [34]. The novel copper catalyst was fully characterized by X-ray crystallographic analysis, UV–visible absorption and emission as well as cyclic voltammetry. The catalyst [Cu(I)(N^N)(DPEPhos)]PF_6_ was used in the chlorotrifluoromethylation of alkenes using CF_3_SO_2_Cl as the ATRA reagent. The reaction was carried out under blue light irradiation (450 nm). The methodology was applied to a broad range of substrates, including (α-)styryl derivatives, cyclic derivatives, acrylates, enol acetates, and enamines. The functional group tolerance of the transformation was fairly decent and demonstrated the synthetic utility of this reaction manifold. To explain the reaction outcome, the authors suggested the following mechanism: The excited copper catalyst reduces the CF_3_SO_2_Cl reagent, resulting in the formation of a sulfonyl radical, which, after elimination of SO_2_, generates the trifluoromethyl radical. This radical then reacts with the olefin to form a carbon-centered radical. Then, two plausible pathways were suggested: The first one involved an oxidation of this carbon-centered radical by the Cu(II) complex, followed by trapping of the carbocation with the chloride anion initially generated in the first reduction step. Alternatively, the authors suggested a possible mechanism involving a radical propagation.

**Scheme 19:**
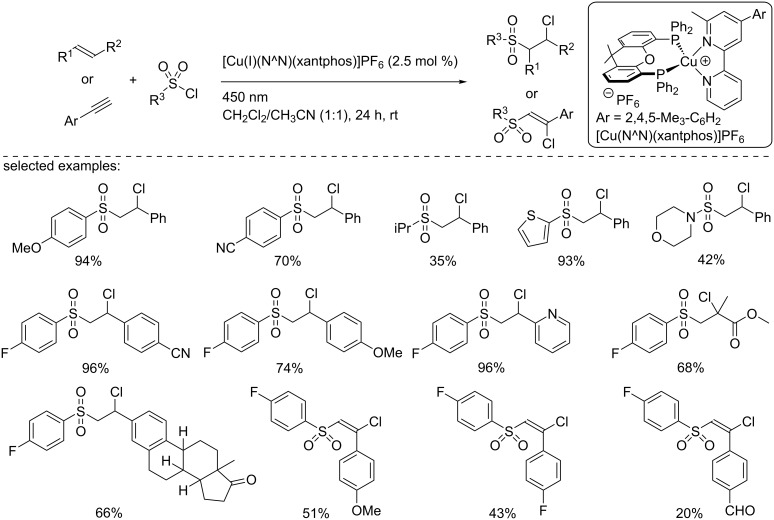
Chlorosulfonylation of styrene and alkyne derivatives by ATRA reactions.

In another report, Hu and co-worker developed a chlorosulfonylation of terminal alkenes and alkynes using the previously developed copper-based photocatalyst (Scheme 19) [35]. The reaction was tolerant to a broad range of arylchlorosulfonyl derivatives when the reaction was carried out with styrene, including sterically hindered and heteroaromatic substrates. Aliphatic sulfonyl chlorides were also tolerated, although the yields were slightly lower. With respect to the alkenes, the reactions proceeded nicely with styrene derivatives as well as with α- and β-substituted styrenes and methyl methacrylate. However, aliphatic substituted alkenes were not suitable substrates or provided the desired products in very low yields (<10%). Finally, the reaction was extended to phenylacetylene derivatives, and the chlorosulfonylated alkenes were isolated in moderate yields.

#### 2.2 Reduction reactions

**Scheme 20:**
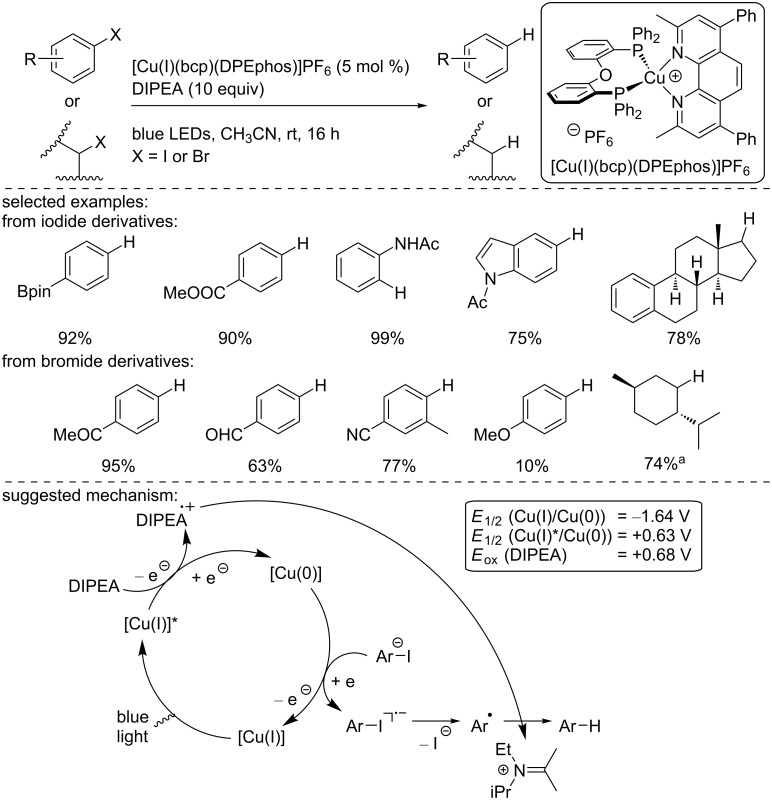
Reduction of aryl and alkyl halides with the complex [Cu(I)(bcp)(DPEPhos)]PF_6_. ^a^Irradiation was carried out at 420 nm. All redox potential are reported vs SCE.

In 2017, Evano and co-workers reported the photocatalytic reduction of aryl and alkyl iodides and bromides catalyzed by a heteroleptic copper complex, [Cu(I)(bcp)(DPEPhos)]PF_6_, under blue light irradiation (Scheme 20) [36]. The reduction of aryl iodides proceeded well, regardless of the electron density of the aromatic ring, and the products were isolated in good to excellent yield, with an excellent functional group tolerance. Moreover, a complex steroid derivative was efficiently reduced under the standard conditions. Activated aryl bromides were also readily reduced under similar conditions, even if they were tedious electron-rich aryl bromide substrates, giving low yield, probably due to their intrinsic reduction potential. Interestingly, the bromide substrate derived from menthol was readily reduced in an excellent 74% isolated yield. The authors conducted some mechanistic studies (including cyclic voltammetry and Stern–Volmer quenching experiments, for instance), and, based on their findings, suggested the following mechanism: After irradiation under blue light, the excited [Cu(I)]* complex is reduced by the organic base DIPEA to produce a [Cu(0)] complex. The latter undergoes an SET with the aryl iodide to generate the radical anion from aryl iodide, which collapses into the aryl radical while the photocatalyst in the ground state is regenerated. Finally, the aryl radical abstracts a hydrogen atom from the oxidized DIPEA to produce the reduced aryl derivative.

**Scheme 21:**
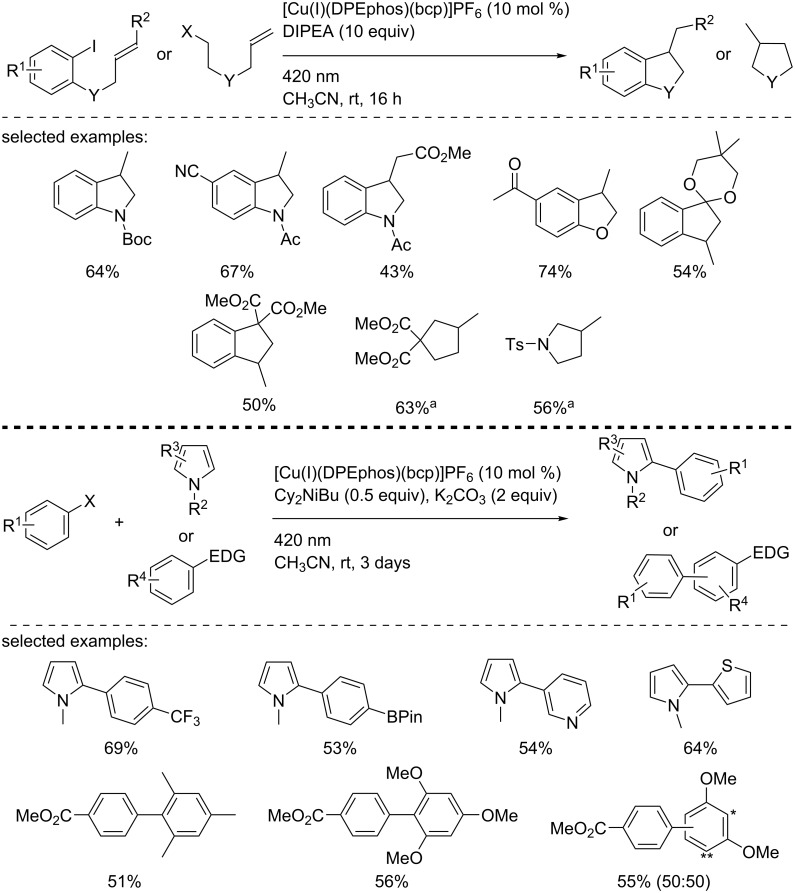
Meerwein arylation of electron-rich aromatic derivatives and 5-*exo*-*trig* cyclization catalyzed by the photocatalyst [Cu(I)(bcp)(DPEPhos)]PF_6_. ^a^5 mol % of catalyst and 5 equiv of DIPEA were used.

Within the same study, Evano and co-workers took advantage of the developed photocatalytic reduction of aryl iodides and bromides into the corresponding aryl radical to use the latter in further transformations (Scheme 21). First, a 5-*exo*-*trig* cyclization was carried out to access indolines, dihydrobenzofurans, indanes, cyclopentane, and pyrrolidines. The cyclized products were isolated in good to excellent yields. Finally, a Meerwein arylation reaction was developed through the copper photocatalyzed formation of an aryl radical according to a reductive process. A large panel of aryl iodides was added to various pyrroles and electron-rich aromatic derivatives. The arylated products were obtained in moderate to good yields, and the functional group tolerance was excellent.

**Scheme 22:**
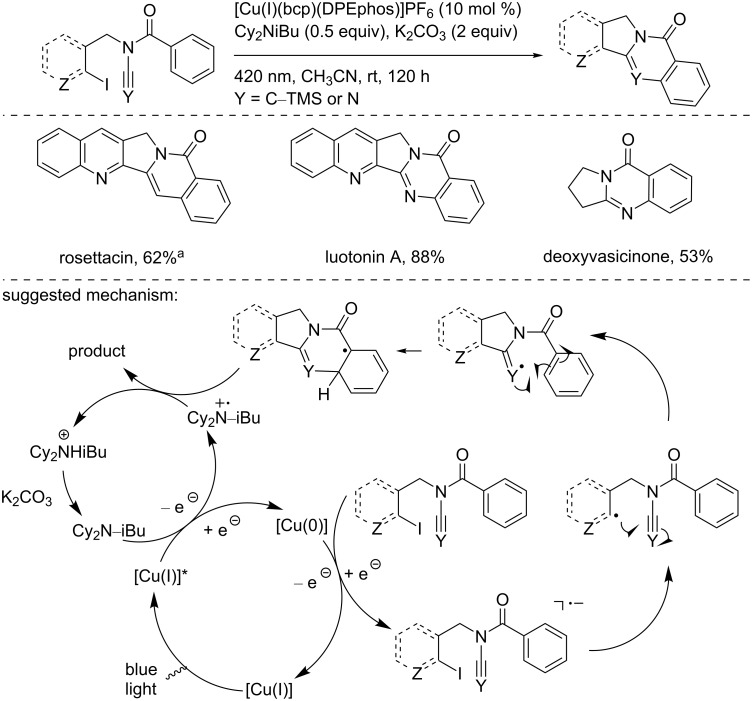
[Cu(I)(bcp)(DPEPhos)]PF_6_-photocatalyzed synthesis of alkaloids. ^a^Yield over two steps (cyclization and TMS deprotection).

Later in 2018, Evano and co-workers used their methodology to reduce aryl iodide for the synthesis of the alkaloids rosettacin, luotonin A, and deoxyvasicinone (Scheme 22) [37]. The developed strategy relied on the addition of an aryl radical on ynamides or cyanamides, followed by a final cyclization. Using similar reaction conditions as those used for the Meerwein arylation, the three alkaloids were synthesized in good to excellent yield in a straightforward manner.

**Scheme 23:**
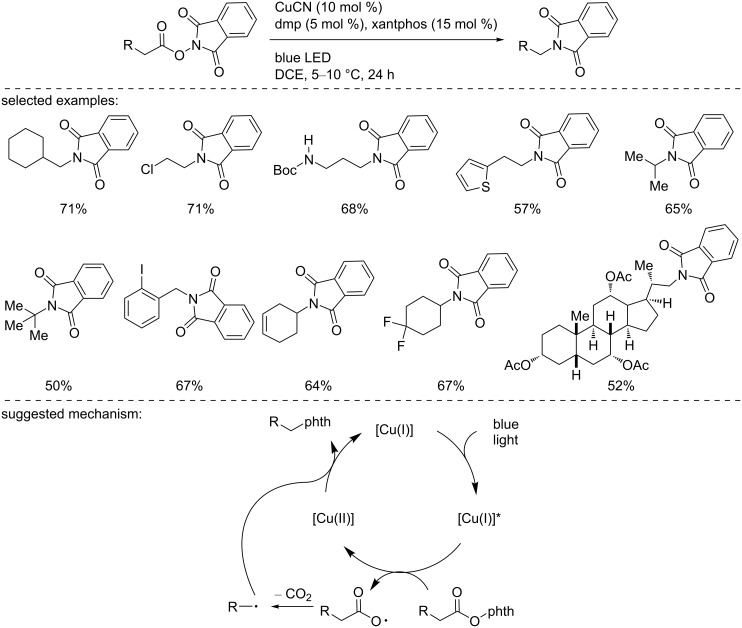
Copper-photocatalyzed decarboxylative amination of NHP esters.

In 2017, Fu, Peters, and co-workers reported the copper-photocatalyzed intramolecular decarboxylative C–N coupling of NHP esters using an in situ-formed heteroleptic copper complex (Scheme 23) [38]. This protocol, a versatile alternative to the Curtius rearrangement, was applied to a large variety of substrates, including primary/secondary alkyl, cycloalkyl, and benzyl NHP esters. The products were obtained in good yields. The functional group tolerance of the process was excellent and was further demonstrated in the course of a robustness screen. Supported by mechanistic experiments, including UV–vis analysis, crossover studies, trapping with TEMPO, and the use of a radical clock, the authors suggested the following mechanism, although the nature of the heteroleptic copper complex has not been clearly determined. However, the involvement of [Cu(I)(dmp)(xantphos]BF_4_ was precluded, since the preformed complex proved to be inefficient under the standard reaction conditions. The authors suggested first the photoexcitation of a [Cu(I)] species. Then, the excited [Cu(I)]* species reduces the NHP ester to form a carboxyl radical and the phthalimide anion, which binds to the [Cu(II)] species. After the elimination of CO_2_, the recombination of the alkyl radical with the [Cu(II)] species bearing the phthalimide forms the product and regenerates the active [Cu(I)] catalyst.

**Scheme 24:**
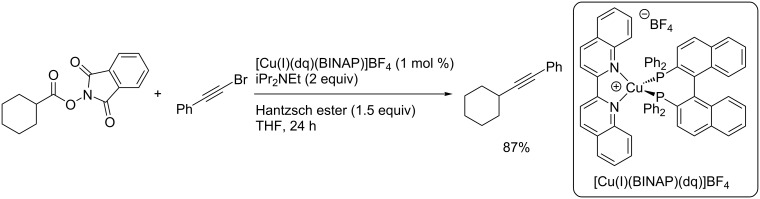
Photocatalytic decarboxylative alkynylation using [Cu(I)(dq)(binap)]BF_4_.

Collins and co-workers described the use of [Cu(I)(dq)(binap)]BF_4_ as an efficient catalyst for the reductive decarboxylative coupling of a NHP ester derived from cyclohexanecarboxylic acid with a bromoalkyne (Scheme 24) [39]. The catalyst was selected using a combinatorial approach for the selection of the optimal catalyst structure. The product was isolated in an excellent 87% yield.

**Scheme 25:**
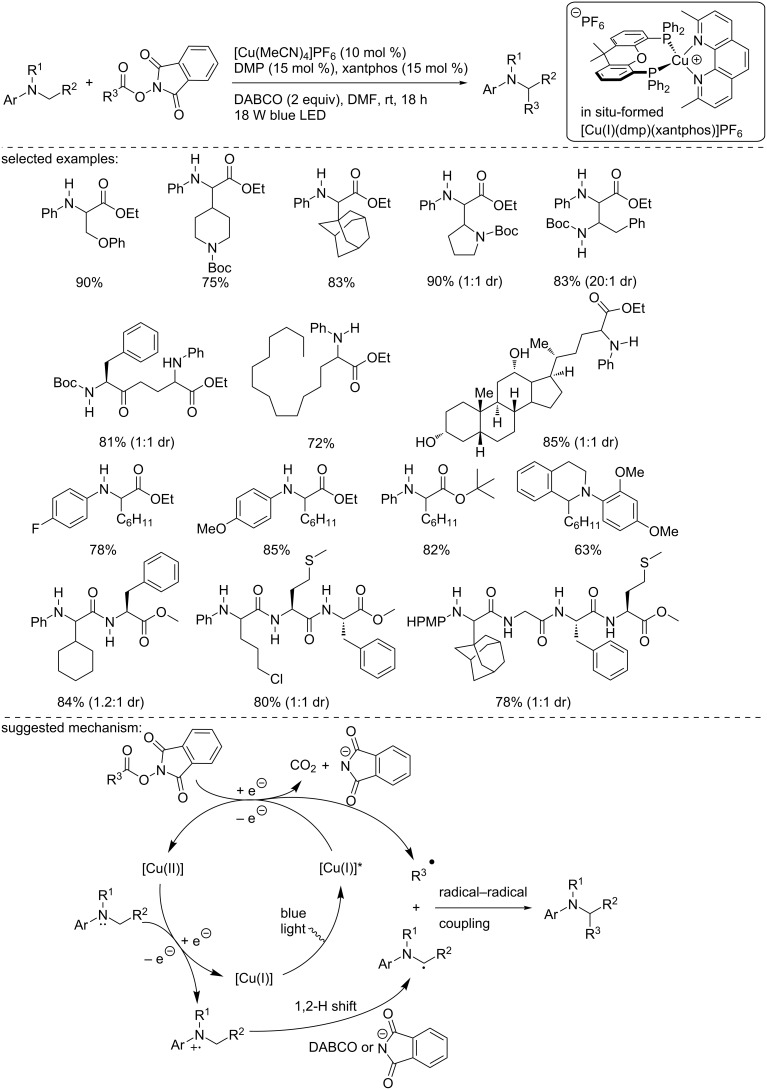
Copper-photocatalyzed alkylation of glycine esters.

In 2018, Wang, Xu, and co-workers described the reductive decarboxylative alkylation of glycine and glycine-containing peptides using an in situ-formed heteroleptic copper complex, [Cu(I)(dmp)(xantphos)]PF_6_ (Scheme 25) [40]. Under blue light irradiation, glycine esters were readily alkylated using NHP esters in the presence of DABCO as a base. A large panel of NHP esters was introduced to ethyl *N*-phenylglycine ester, and the alkylated products were obtained in good to excellent yields. In addition, various glycine derivatives were also alkylated in good yields. Finally, the authors demonstrated the synthetic interest of their methodology through the alkylation of di- and tripeptides. The authors studied the reaction mechanism and suggested the following one: First, the excited in situ-formed copper complex reduced the NHP ester, as demonstrated by the Stern–Volmer quenching experiment. The formed radical anion collapsed into the corresponding alkyl radical and the phthalimide anion. Then, the oxidized copper complex oxidized the glycine ester, regenerating the catalyst, furnishing the N-centered radical cation. Then, the latter underwent a 1,2-hydride shift in the presence of the base (or the phthalimide anion) to form the α-amino radical that recombined with the alkyl radical formed in the initial step.

**Scheme 26:**
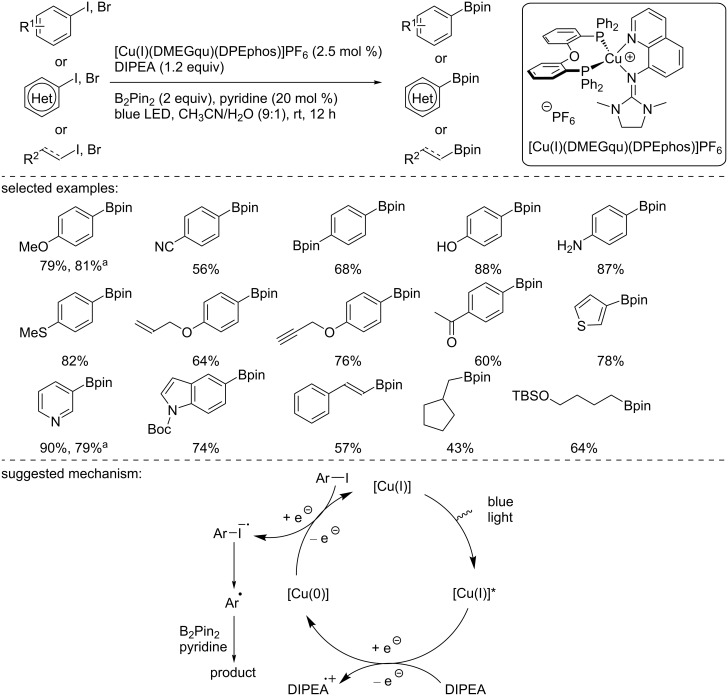
Copper-photocatalyzed borylation of organic halides. ^a^Under continuous flow conditions.

In early 2019, our research group reported the copper-photocatalyzed borylation of organic halides using [Cu(I)(DMEGqu)(DPEPhos)]PF_6_ as the photocatalyst (Scheme 26) [41]. The photocatalytic Miyaura borylation reaction was carried out using aryl iodides bearing either electron-donating or electron-withdrawing groups in good to excellent yields. Activated aryl bromides and heteroaryl bromides were also successfully converted to the corresponding arylboronic esters in good to excellent yields. The methodology was also extended to a set of vinyl iodides, providing the vinylboronic esters in moderate to good yields. Notably, alkyl iodides were also suitable substrates. The methodology proved to be compatible with a broad range of functional groups. To demonstrate the synthetic utility of the process, the reaction was developed under continuous flow conditions to afford an easy and straightforward scale-up of the method (up to 5 g). Mechanistic studies were carried out, suggesting a possible [Cu(I)]/[Cu(I)]*/[Cu(0)] reaction manifold. The copper photocatalyst in the excited state is reduced to the [Cu(0)] complex by the sacrificial organic reductant DIPEA. The in situ-formed [Cu(0)] species reduces the organic halide to generate the corresponding radical anion, which loses the halide anion and gives rise to the corresponding C-centered radical. A subsequent trapping with B_2_Pin_2_ affords the borylated product.

**Scheme 27:**
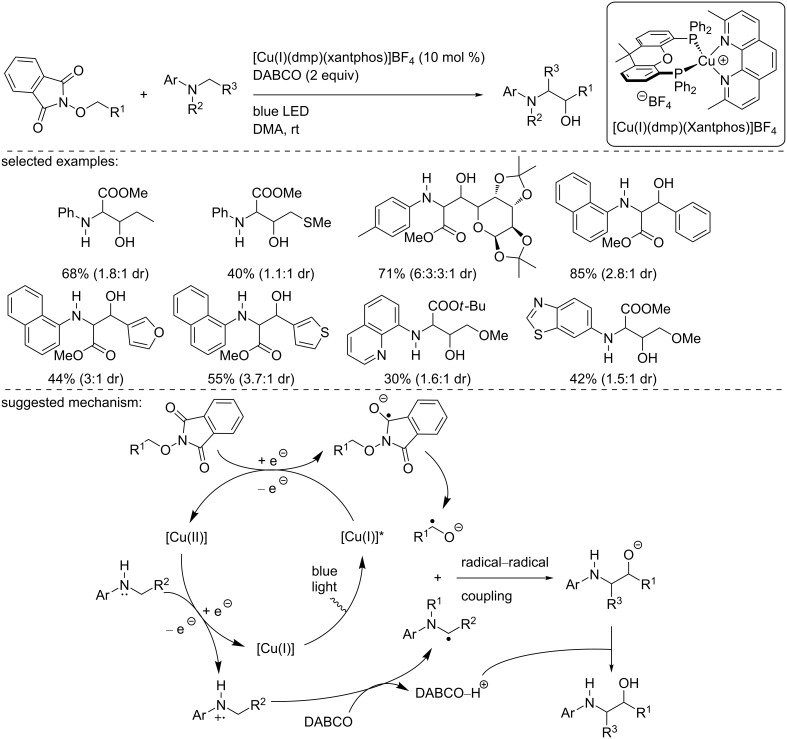
Copper-photocatalyzed α-functionalization of alcohols with glycine ester derivatives.

In 2019, Wang, Duan, and co-workers reported the reductive α- and δ-functionalization of alcohol using *N*-alkoxyphthalimide derivatives and glycine esters under basic or acidic conditions, respectively [42]. With regards to the α-functionalization, various *N*-arylated glycine esters were used as reaction partners with a broad range of *N*-alkoxyphthalimide derivatives, using [Cu(I)(dmp)(xantphos)]BF_4_ as the catalyst and DABCO as the base (Scheme 27). The products were obtained in moderate to good yields and moderate diastereoselectivities. The reaction was applied to *N*-alkoxyphthalimides derived from aliphatic and benzyl alcohols and heteroaromatic ones. To explain the reaction outcome, the authors suggested the reduction of the *N*-alkoxyphthalimide by the excited copper catalyst, [Cu(I)]*. Then, this radical collapses into an α-oxy carbon-centered radical thanks to an intramolecular proton transfer. This carbon-centered radical then recombines with the α-amino radical that arises from the oxidation of the amine with the formed [Cu(II)] complex, followed by a deprotonation by DABCO. The resulting alkoxide is finally converted into the alcohol by the protonated DABCO.

**Scheme 28:**
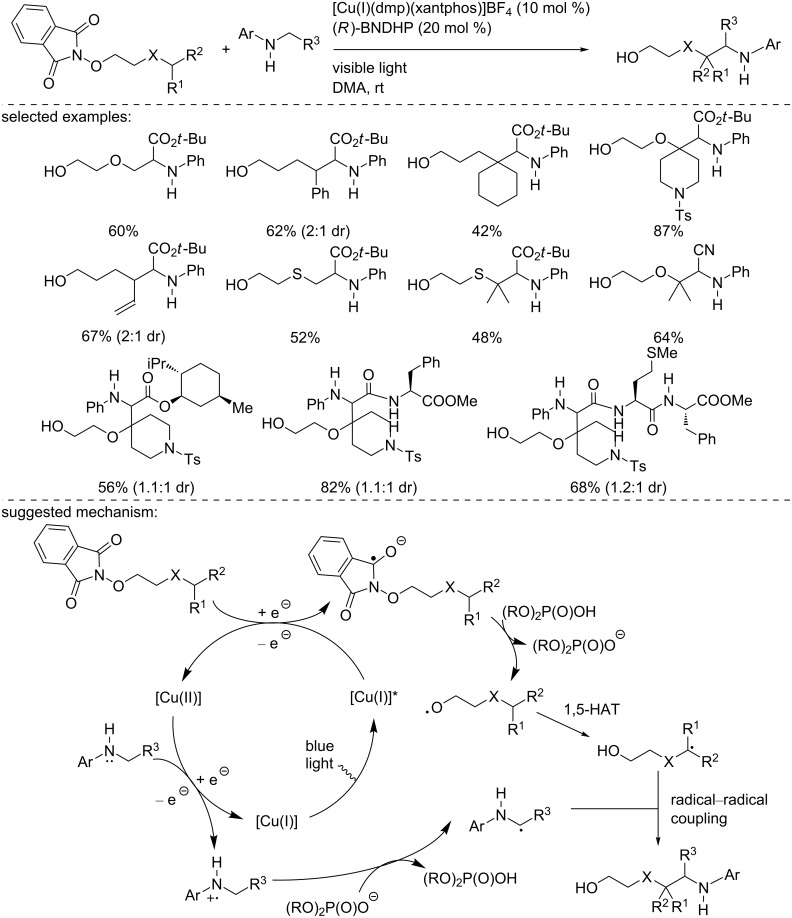
δ-Functionalization of alcohols using [Cu(I)(dmp)(xantphos)]BF_4_.

During this study, the authors found that replacement of the base by the Brønsted acid (*R*)-1,1-binaphtyl-2,2-diyl hydrogen phosphate ((*R*)-BNDHP) under similar reaction conditions and using the same copper catalyst provided the δ-functionalization of the alcohol (Scheme 28). This reaction manifold was applied to a large variety of *N*-alkoxyphthalimide derivatives using glycine ester derivatives and was also extended to more complex structures, including di- and tripeptides. These examples demonstrated the synthetic utility of the methodology. The reaction pathway was explained as follows by the authors: Similarly to the α-functionalization, the excited copper photocatalyst reduces the *N*-alkoxyphthalimide. Then, in the presence of the acid, it is protonated and collapses into the corresponding alkoxy radical. This radical carries out a 1,5-HAT to generate a carbon-centered radical. Finally, this radical recombines with the α-amino radical generated from the above-mentioned pathway.

#### 2.3 Oxidation reactions

**Scheme 29:**
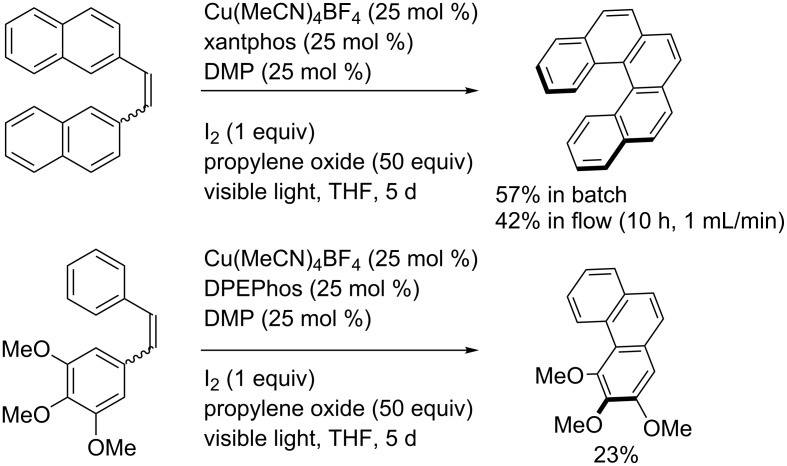
Photocatalytic synthesis of [5]helicene and phenanthrene.

In 2012, Collins and co-workers described a copper-catalyzed photocyclization to synthesize [5]helicene (Scheme 29) [43]. Using the in situ-formed [Cu(I)(dmp)(xantphos)]BF_4_ (25 mol %) in the presence of iodine and propylene oxide as the oxidant system under visible light irradiation, [5]helicene was synthesized in 57% yield. Importantly, the reaction scale was increased to 1 g under continuous flow conditions. Then, the reaction was extended to the cyclization of a stilbene derivative into phenanthrene in a moderate 23% isolated yield. To explain the reaction pathway, the authors suggested an oxidative mechanism.

**Scheme 30:**
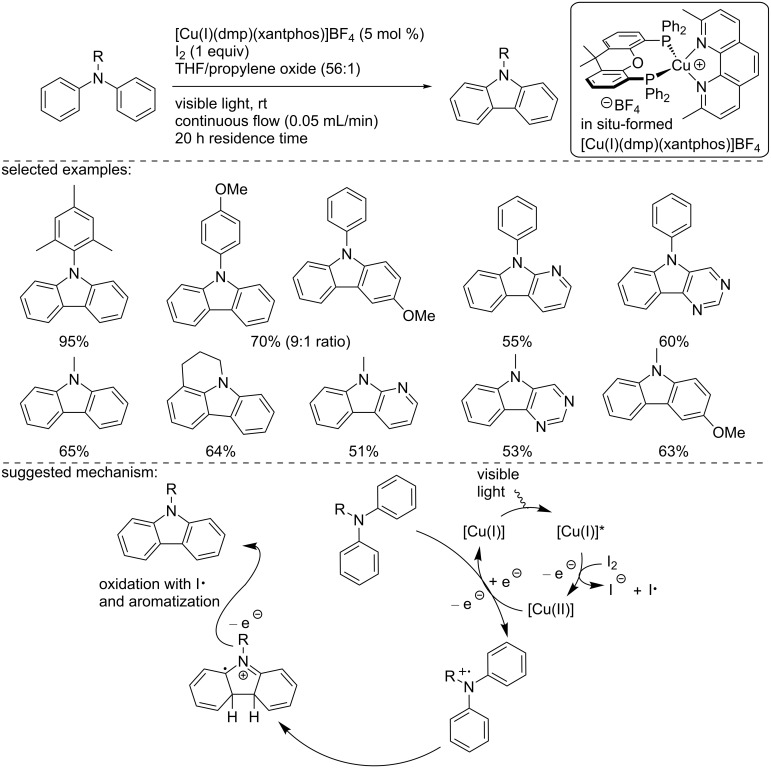
Oxidative carbazole synthesis using in situ-formed [Cu(I)(dmp)(xantphos)]BF_4_.

One year later, 2013, Collins and co-workers extended this concept to the synthesis of carbazole derivatives under continuous flow conditions (Scheme 30) [44]. Using, the [Cu(I)(dmp)(xantphos)]BF_4_ catalyst, the oxidative cyclization of various triarylamines was carried out in the presence of I_2_ and propylene oxide as a proton scavenger under flow conditions (0.05 mL/min; residence time = 20 h). The use of flow conditions drastically enhanced the kinetics of the reaction, moving from 120 h reaction time in batch to 20 h using a continuous flow reactor. The reaction allowed the synthesis of a large panel of carbazoles. The selectivity of the reaction using an unsymmetrical triarylamine depended on the electronic density of the aromatic ring, furnishing, in some cases constitutional isomers. Importantly, in the case of heteroaryl-substituted triarylamines, the heterocycle was incorporated in the carbazole structure, and a single isomer was formed. Then, the methodology was extended to *N*-alkyldiarylamines, the products were isolated in good yields, and no dealkylation was observed for *N*-methyldiarylamines. To explain the reaction outcome, the authors suggested a mechanism involving oxidative quenching. First, the excited [Cu(I)]* species was oxidized in the presence of iodine, furnishing a [Cu(II)] complex. Then, an oxidation of the diarylamine occurred, generating the N-centered radical cation, which undergoes an intramolecular cyclization. A final oxidation of the aryl radical intermediate with an iodine radical, followed by aromatization, generated the desired carbazole.

**Scheme 31:**
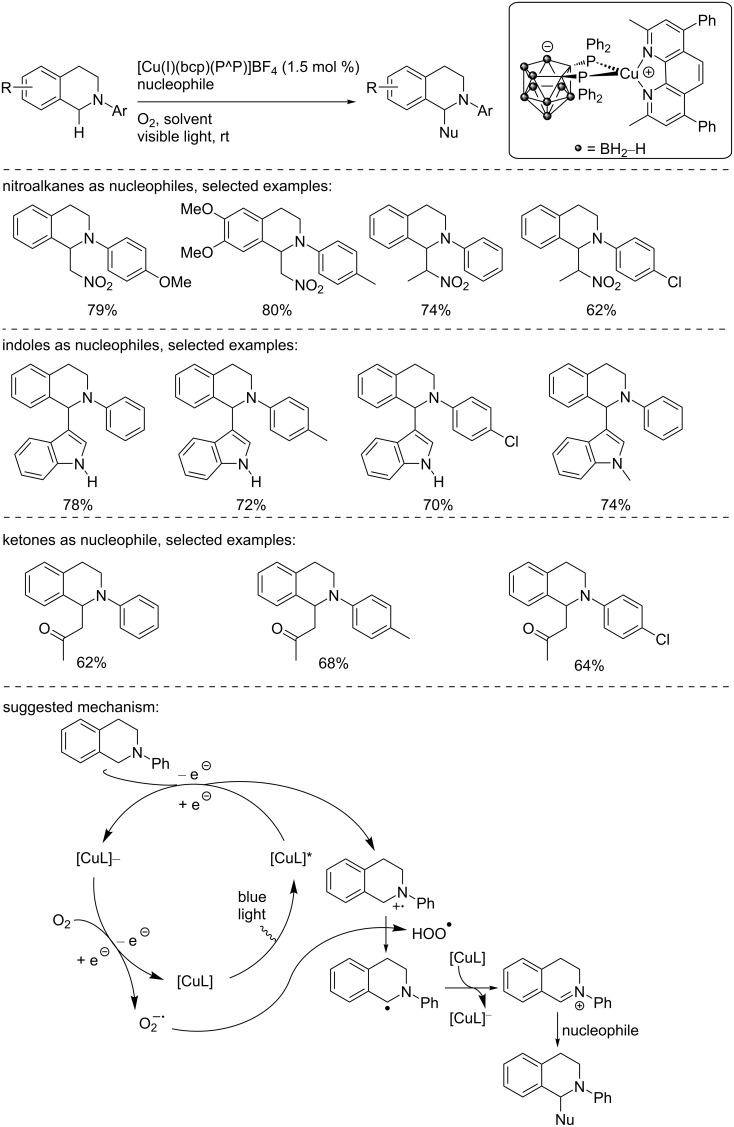
Copper-photocatalyzed functionalization of *N*-aryl tetrahydroisoquinolines.

Later in 2015, the group of Che described the synthesis of a zwitterionic copper(I) complex having a phenanthroline ligand (bcp) and a *nido*-carborane-diphosphine ligand. This complex was used in a benchmark reaction in photocatalysis, the oxidation of *N*-aryl tetrahydroisoquinoline (Scheme 31) [45]. Upon oxidation, the in situ-formed iminium ion was reacted with nitroalkanes, enamines, and indoles. In all cases, the 1-substituted tetrahydroisoquinolines were isolated in good to excellent yields. The reaction mechanism suggested by the authors was in agreement with the literature [46]: The excited copper catalyst oxidizes the tetrahydroisoquinoline and forms an N-centered radical cation. The copper catalyst is regenerated upon oxidation with O_2_. The resulting superoxide radical anion allowed the formation of the α-amino radical that is then oxidized to the dihydroisoquinolinium species. Then, the latter reacted with the nucleophile (nitroalkanes, catalytically in situ-formed enamines and indoles) to furnish the product.

#### 2.4 Proton-coupled electron transfer (PCET)

The PCET reaction is an interesting and unusual sequence to functionalize molecules. Indeed, it relies on a redox transformation with a concerted proton and electron exchange. Note that this process can occur either through an oxidative or a reductive pathway [47–49].

**Scheme 32:**
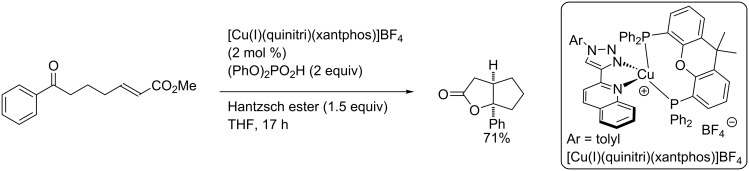
Bicyclic lactone synthesis using a copper-photocatalyzed PCET reaction.

In 2018, during the development of a combinatorial approach to select the best copper-based photocatalyst, Collins and co-workers reported the synthesis of a bicyclic lactone by a PCET reaction manifold (Scheme 32) [39]. Using their methodology, the authors discovered the [Cu(I)(quinitri)(xantphos)]BF_4_ catalyst as the best one, which afford the *cis*-lactone in 79% isolated yield.

**Scheme 33:**
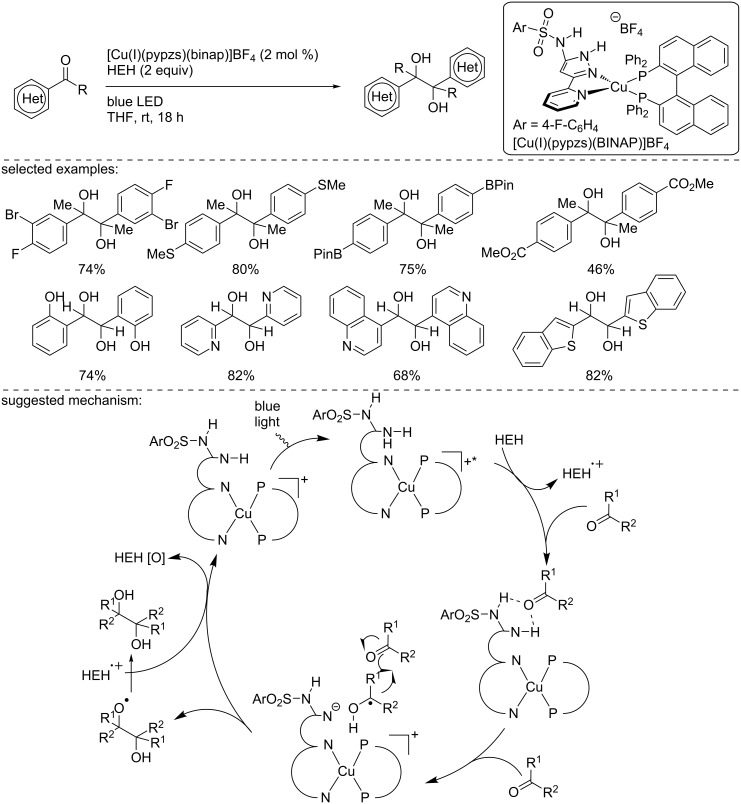
Photocatalytic Pinacol coupling reaction catalyzed by [Cu(I)(pypzs)(BINAP)]BF_4_. The ligands of the copper complex are omitted for clarity.

In 2019, Collins and co-workers reported the synthesis of a new bifunctional copper photocatalyst for the reductive pinacol coupling of ketones and aldehydes (Scheme 33) [50]. This new catalyst was designed to activate the ketones or aldehydes due to an acidic proton present on the structure and to carry out an SET when excited by light. This approach represents the first report describing the design of a Cu-based photocatalyst able to carry out an SET and an activation of the substrate, in this case through H-bonding. This close proximity increased the yield of the transformation and avoided the presence of a Brønsted acid in the reaction media. As such, Collins and co-workers developed the catalyst [Cu(I)(pypzs)(BINAP)]BF_4_ where the ligand (5-(4-fluorosulfonyl)amino-3-(2pyridyl)pyrazole)) (pypzs) had an acidic proton prone to activate the carbonyl group during the pinacol coupling reaction. In the presence of 2 mol % of the catalyst, the Hantzsch ester (HEH), as a hydrogen atom donor, under blue light irradiation, a large panel of ketones and aldehydes was readily converted into the corresponding 1,2-diols in moderate to excellent yields. The functional group tolerance of the reaction was excellent as bromides, phenols, thioethers, esters, boronic esters, and heterocycles, including pyridine and quinolines, were well tolerated. The authors carried out mechanistic studies and demonstrated the H-bonding ability of their catalyst by NMR studies. The following mechanism was suggested to explain the reaction pathway: After irradiation, the copper catalyst, in an excited state, is quenched by the HEH, as demonstrated by a quenching experiment. Then, the reduced catalyst is involved in a hydrogen bond interaction with the carbonyl derivative, attributed to the acidic proton present on the pypzs ligand, and activates the carbonyl group. The PCET occurs, furnishing a ketyl radical that can react with another molecule (ketone or aldehyde), delivering the pinacol product. A final HAT with another equivalent of HEH or HEH^+^ delivers the product, and the resulting radical cation of the Hantzsch ester allows the regeneration of the catalyst in the ground state.

#### 2.5 Reaction based on an energy transfer (EnT)

Reactions based on energy transfer represent an interesting class of photocatalyzed transformations. Indeed, this reaction sequence relies on the deactivation of an excited molecule through a transfer of energy to a second one. Then, this second molecule is raised to the excited state and can then react [51–52].

**Scheme 34:**
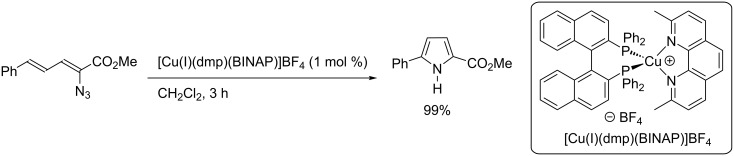
Azide photosensitization using a Cu-based photocatalyst.

During the course of the design of a library of copper photocatalysts through a combinatorial approach, Collins and co-workers reported the copper-photocatalyzed vinyl azide sensitization to allow the formation of the corresponding 2,5-disubstituted pyrrole (Scheme 34) [39]. The reaction was promoted by a visible-light irradiation (450 nm) using the complex [Cu(I)(dmp)(BINAP)]BF_4_, and the desired pyrrole was obtained in quantitative yield.

## Conclusion

Over the last decade, photocatalysis using homoleptic or heteroleptic copper complexes experienced a significant growth. The use of these inexpensive and readily available complexes in a broad range of applications, as discussed in this review, clearly demonstrate the growing importance of these catalysts. We do believe that the field of copper photocatalysis using well-defined complexes is still in its infancy, and for sure, new and unexpected reactivities will be discovered in a near future by using these catalysts.
